# 1298. Donor Call Simulation: A Novel Medical Education Tool to Evaluate Trainees’ Clinical Decision-Making in Transplant Infectious Disease

**DOI:** 10.1093/ofid/ofac492.1129

**Published:** 2022-12-15

**Authors:** Rachel Sigler, Darcy Wooten, Rebecca Kumar, Jonathan Hand, Nicholas Marschalk, Roderick Go, Katya Prakash, Erica J Stohs, Nancy Law

**Affiliations:** University of Pittsburgh, San Diego, California; University of California, San Diego, San Diego, California; Georgetown University Medical Center, Washington, District of Columbia; Ochsner Health, New Orleans, Louisiana; The Ohio State University Medical Center, Columbus, Ohio; Stony Brook University Hospital, Stony Brook, New York; University of Maryland, Baltimore, Maryland; University of Nebraska Medical Center, Omaha, Nebraska; University of California, San Diego, San Diego, California

## Abstract

**Background:**

Simulation is a useful education tool for high-stakes clinical skills and decision-making. Recommending whether to accept or reject an organ for transplantation based on infection risk is a critical core competency in Transplant Infectious Disease (ID), however there are no published data that learners have opportunities to practice this during training. We created a novel simulation to expose learners to this real-life clinical scenario and evaluated their clinical decision-making in these situations.

**Methods:**

We created 6 simulations with common ID consult questions about whether to accept or reject an organ for transplant based on infection risk (Table 1). During learners’ Transplant ID rotations, faculty periodically texted or paged them with the simulation cases as though they were the transplant coordinator. Learners had 15 minutes to ask follow up questions before deciding to accept or reject the organ and explain their decision-making process in a survey. Learners completed a survey 1 month after the simulation experience to evaluate its effectiveness.

**Figure:**
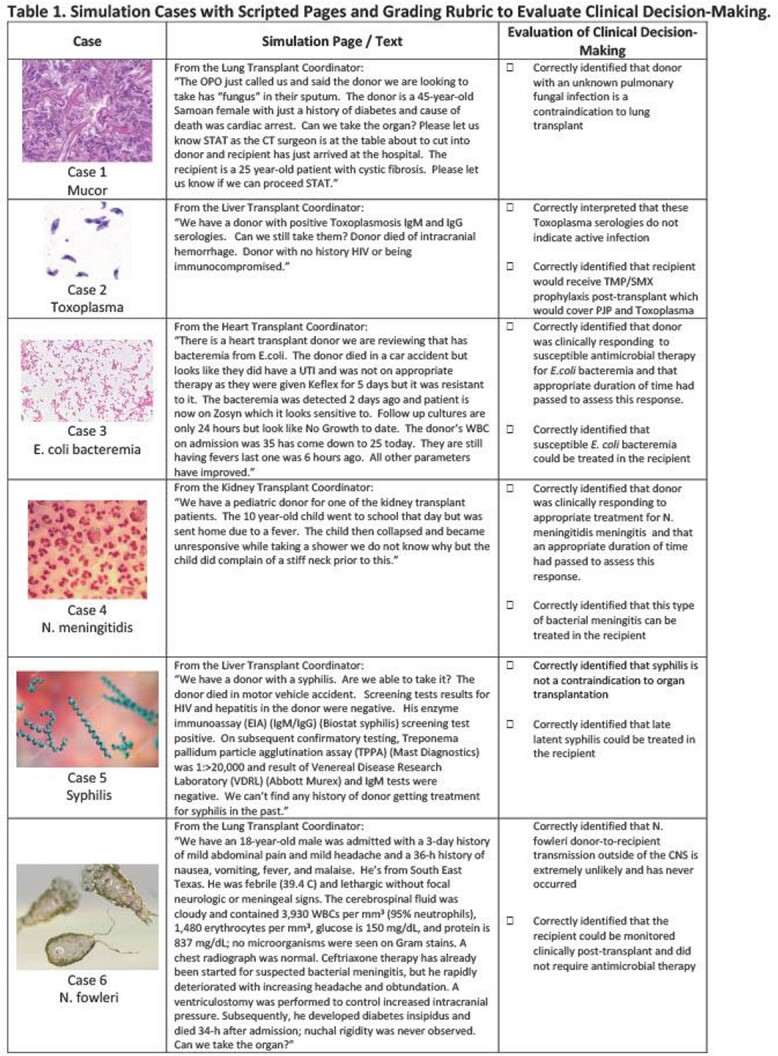

**Results:**

Between October 2021 and April 2022, 16 learners from 7 medical centers participated in the simulation (Table 2) and 94% (15/16) completed the follow up survey. Eighty-seven percent (13/15) of ID learners reported that the simulation was effective in teaching them when to accept or reject organs and 80% (12/15) felt more prepared to make these decisions in practice. Most learners correctly identified acceptable organs for transplant during the simulations (Figure 1). Of the 100 clinical reasoning decisions made during the activity, 19% were discordant, where the learner correctly decided to accept or decline the organ but with incorrect or incomplete reasoning for this decision (Figure 2).

**Figure:**
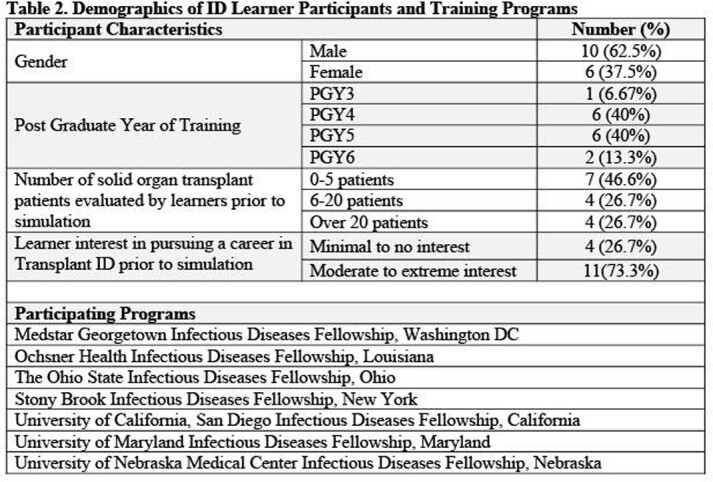

**Figure:**
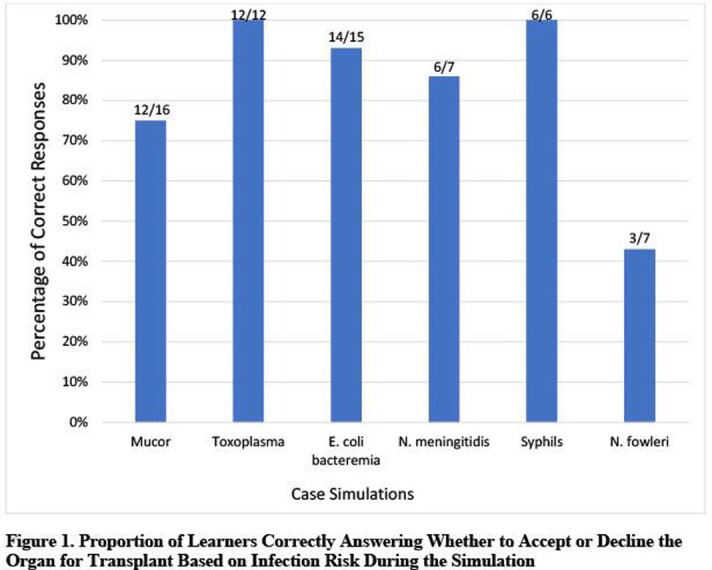

**Figure:**
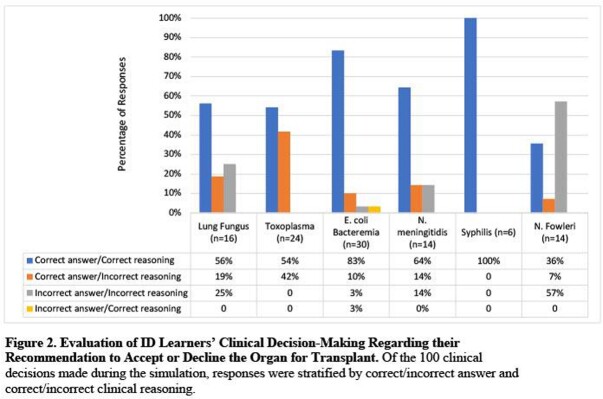

**Conclusion:**

ID learners perceived our transplant ID simulation as an effective educational tool to learn when to accept or reject an organ for transplant. By evaluating the clinical reasoning behind these decisions our simulation provides ID educators with nuanced insight into their learners' thought process and allows for targeted coaching to correct deficits in reasoning.

**Disclosures:**

**Rebecca Kumar, MD**, Gilead: Grant/Research Support|Regeneron: Grant/Research Support **Jonathan Hand, MD**, GlaxoSmithKline: Grant/Research Support|Janssen: Grant/Research Support|Pfizer: Grant/Research Support **Roderick Go, DO**, Aptose Biosciences: Stocks/Bonds|Bristol Meyers Squibb: Stocks/Bonds|Cytodyn Inc.: Stocks/Bonds|Scynexis: Grant/Research Support **Erica J. Stohs, MD, MPH**, bioMerieux: Grant/Research Support.

